# Differential neuropathic pain sensitivity and expression of spinal mediators in Lewis and Fischer 344 rats

**DOI:** 10.1186/1471-2202-15-35

**Published:** 2014-02-28

**Authors:** Glenn-Marie Le Coz, Cathy Fiatte, Fernand Anton, Ulrike Hanesch

**Affiliations:** 1 Laboratory of Neurophysiology & Psychobiology, University of Luxembourg, 162a, avenue de la Faïencerie, Luxembourg, L-1511, Luxembourg

**Keywords:** HPA axis, Rat strains, Chronic constriction injury, Glutamate transporters, Glia cells, Spinal cord

## Abstract

**Background:**

Altered hypothalamo-pituitary-adrenal (HPA) axis activity may be accompanied by a modulation of pain sensitivity. In a model of neuropathic pain (chronic constriction injury, CCI) we investigated the onset and maintenance of mechanical allodynia/hyperalgesia and the expression of biochemical mediators potentially involved in spinal cell modulation in two rat strains displaying either hypo- (Lewis-LEW) or hyper- (Fischer 344-FIS) reactivity of the HPA axis.

**Results:**

Mechanical pain thresholds and plasmatic corticosterone levels were assessed before and during periods of 4 or 21 days following CCI surgery. At the end of the respective protocols, the mRNA expression of glial cell markers (GFAP and Iba1) and glutamate transporters (EAAT3 and EAAT2) were examined. We observed a correlation between the HPA axis reactivity and the pain behavior but not as commonly described in the literature; LEW rats seemed to be less sensitive than FIS from 4 to 14 days after the CCI surgery when looking at the mechanical allodynia/hyperalgesia. However, the biochemical spinal markers expression we observed is conflicting.

**Conclusion:**

We did not find a specific causal relation between the pain behavior and the glial cell activation or the expression of the glutamate transporters, suggesting that the interaction between the HPA axis and the spinal activation pattern is more complex in a context of neuropathic pain.

## Background

Neuropathic pain is the consequence of a lesion or a disease affecting the central and/or the peripheral nervous system [1]. It is a major and common health issue remaining difficult to treat [2].

Clinical studies have shown that many patients suffering from functional pain syndromes like fibromyalgia [3], migraine and tension headache [4], trigeminal neuralgia [5], low-back pain [6] or rheumatic arthritis [7] display a reduced hypothalamic-pituitary-adrenal (HPA) axis reactivity. The HPA axis is an interface between the endocrine and the nervous system and plays an important role in the maintenance of homeostasis during stressful events [8]. It has been hypothesized that an alteration of adrenocortical reactivity may lead to an ongoing sensitization of peripheral or central nociceptive neurons [9] and hence to the enhanced pain sensitivity observed in these patients.

Many studies attempted to elucidate the relationship between altered HPA axis activity and nociception through different animal models. Lewis (LEW) and Fischer 344 (FIS) are two histocompatible inbred rat strains presenting different HPA axis reactivity. Relative to other strains, LEW rats exhibit a blunted HPA axis activity due to a reduced synthesis and secretion of corticotropin-releasing hormone (CRH), leading to a decreased release of adrenocorticotropic hormone (ACTH) from the pituitary and corticosterone from the adrenal cortex [10-13]. On the contrary, the HPA axis of FIS rats is hyperresponsive, resulting in a significantly higher blood corticosterone level compared to LEW rats [10]. Basal and stimulated pro-inflammatory cytokine levels also have been shown to be increased in LEW relative to FIS [14]. The two strains display sensitivity discrepancies in response to acute and chronic pain, including neuropathic pain [15-17]. As shown by Fecho and Valtschanoff [16] the lower plasma levels of corticosterone seen in LEW rats correlate with their exacerbated mechanical allodynia exhibited after partial sciatic nerve ligation. However, the development and maintenance of neuropathic pain require a complex network of pathways [18], implying that differences in HPA axis cannot *per se* explain the observed strain-specific differences in neuropathic pain behavior.

The glutamatergic system is known to be part of this pain-controlling network. Glutamate is a major excitatory neurotransmitter whose actions are mediated by ionotropic and metabotropic receptors [18], and is considered as the main pain mediator [19]. After injury, glutamate-induced plasticity plays a key role in the mechanism of activity-dependent central sensitization, thus contributing to the development of post-injury pain hypersensitivity [20]. The role of glutamate in nociception is also dependent on its transporters, the excitatory amino acid transporters (EAATs), which control glutamate clearance and its availability [21-25]. Actually, it has been shown in a chronic constriction injury (CCI) model of neuropathic pain that spinal glucocorticoid receptor activation mediates a downregulation of EAAC1/EAAT3 expression, a neuronal transporter of glutamate, in the spinal cord dorsal horn [26]. As a result, the glutamatergic activity and glutamate-mediated neuronal plasticity would be enhanced and could contribute to the neuropathic pain behavior.

Microglial and astrocytic activation is also involved in the initiation and maintenance of neuropathic pain [27-33]. This activation leads to an increased expression of specific microglial and astrocytic markers such as Iba-1 and GFAP, respectively [34,35] and enhances pain sensitivity *via* the release of pro-inflammatory cytokines and glutamate [36-41].

In light of these studies, genesis and maintenance of neuropathic pain imply the supraspinal and spinal parts of the central nervous system, along with their neuronal and non-neuronal components. In the present study, we focused on the regulation of these different systems at the spinal level in three rat strains displaying different HPA axis reactivity backgrounds. More specifically, we assessed the mechanical allodynia of LEW, FIS and Wistar (WIS) rats undergoing CCI-induced neuropathic pain and measured their plasma corticosterone levels over time. Additionally, we quantified the expression of glutamate transporters (EAAT2 and EAAT3) and astrocytic/microglial activation markers (GFAP and Iba-1, respectively) in the spinal cord of each of the respective rat strains.

## Methods

### Animals

Experiments were performed in 108 adult 50-60 day old male LEW, FIS, and WIS rats (36 animals per strain) weighing 124-184 g. The study was only performed in male rats to avoid potential interactions with estrogens. The animals (Harlan Laboratories, Netherlands) were housed three per cage in a temperature-controlled room (20-22°C) under a 12 h day-night cycle. Food and water were provided *ad libitum*. Starting two weeks before initiation of the experiments the animals were handled daily and habituated to the behavioral testing room and devices. The Animal Care and Use Committee of the University of Luxembourg approved all animal procedures.

### CCI and sham surgery

For CCI surgery, the rats were anesthetized with isoflurane (Isoflo®, Abbott Laboratories Ltd, Berkshire, UK) using an anesthesia unit (Univentor 400, Zejtun, Malta). The right sciatic nerve was exposed and three loose Dexon S (United States Surgical, Tyco Healthcare Group, North Haven, CT, USA) ligatures were placed around the nerve in a distance of about 1 mm. Subsequently, the muscle layer was stitched with 4–0 silk suture and the skin layer was closed with surgical skin staples.

For the sham operation, rats underwent the same surgical procedure but without placing ligatures around the sciatic nerve. These animals were used as controls since our goal was to characterize parameters specifically related to the development and maintenance of neuropathic pain that should be differentiated from effects induced by the surgical procedure per se. We took into account that the respective (control) pain behavior and biochemical processing might also change over the test period by plotting differences for identical time points.

### Experimental protocol

The 36 animals per strain were grouped as follows: 24 rats (12 that received sham operation and 12 that underwent CCI surgery) were taken for behavioral tests and blood sampling. They were sacrificed at the end of the experiment at day 21. Half of the rats (6 sham and 6 CCI per strain) were perfused for further immunohistochemical procedures and the other half (also 6 sham and 6 CCI per strain) were decapitated for mRNA isolation and subsequent qPCR performing. The remaining 12 rats per strain (6 sham and 6 CCI operated) were sacrificed already 4 days after the surgery for isolation of mRNA and subsequent qPCR.

We chose day 4 as the first time point of measurement of biochemical markers since by then acute neuropathy had developed while effects primarily related to surgery should have declined. In addition, the FIS and LEW rats displayed differential levels of pain behavior at that time point. In order to obtain measurements relative to the maintenance of neuropathy, a second set of biochemical data was collected at day 21 when the tested strains still displayed pronounced levels of CCI-related mechanical allodynia.

Mechanical pain thresholds were assessed on 3 consecutive days preceding the operation (days −2, −1 and 0) to reveal baseline values. After the CCI or sham surgery (day 0) further measurements were performed on days 4, 7, 10, 14, and 21.

Blood sampling was carried out on day 0 to reveal baseline corticosterone levels and on days 1, 4, 7, 14, and 21 following CCI surgery.

### Von Frey monofilament test

The von Frey test (OptiHair, MarstockNervTest, Germany) was used to measure mechanical allodynia/hyperalgesia. Rats were placed in Plexiglas® cages with a wire mesh floor and given 10 min to acclimate. Each monofilament was placed perpendicularly onto the midplantar surface of the hind paw and pressure was increased until the point of deflection of the filament was reached. Pain thresholds were determined by the ascending and descending method of limits with forces ranging from 8 to 256 mN in 11 logarithmic steps [42]. Each test was repeated three times and the mean was calculated for each paw.

For each strain the baseline was calculated as mean of days −2, −1, and 0. For a better assessment of the CCI effect in the individual strains we set the baseline as 100% for each animal and expressed the paw withdrawal thresholds of the right affected paw as percentage of baseline. For each time point the mean per strain was computed. The data are presented as mean ± standard error of the mean (s.e.m.).

### Blood sampling and measurement of corticosterone levels

Blood sampling was performed in awake animals between 9 h00 and 9 h30, half an hour before the start of the behavioral tests. To minimize stress reactions, rats were loosely held in a towel. A small incision about 15 mm from the end of the tail was made by the use of a scalpel. 300 μl of blood was collected with a capillary tube coated with EDTA (Microvette CB300). The samples were immediately placed on ice, then centrifuged at 4°C for 10 min and the plasma was removed and stored at −20°C. Serum corticosterone levels were measured using an ELISA (Enzyme-Linked Immunosorbent Assay) kit (Assay Designs, USA). The analysis was performed according to the manufacturer’s protocol. Data were acquired via a plate reader (Sunrise Magellan, Austria) and an adequate software package (Magellan software, v6.4 standard, Austria).

### Tissue preparation for immunohistochemistry

At day 21, the 12 rats per strain (6 CCI and 6 sham operated animals) that were planned for protein quantification by immunohistochemistry were deeply anesthetized with an intraperitoneal injection of Penthotal (100 mg/kg, Abbott Laboratories, IL, USA). Initially a solution of tyrode (pH 7.3) containing heparin (Heparin-Natrium Braun, 10,000 I.E./ml, 2 ml/l) was perfused transcardially followed by Zamboni’s fixative consisting of 4% paraformaldehyde and 0.2% picric acid in 0.1 M phosphate-buffered saline (PBS; pH 7.4). Spinal cord segments L4 and L5 were removed and post-fixed in the same fixative during at least 2 h. Following cryoprotection in 30% sucrose in 0.1 M phosphate buffer (PB) at 4°C for 2 h, 30 μm sections of spinal cord were cut using a cryostat and mounted on microscope slides (10–15 sections per slide).

### Immunohistochemistry

The spinal cord sections were washed in PBS and incubated in 0.1% H_2_O_2_ to inactivate endogenous peroxidases. Then they were rinsed in three changes of PBS and subsequently preincubated during 30 min in 5% normal serum in PBS containing 0.5% Triton X-100. Afterwards, the sections were incubated overnight at room temperature with either anti-GFAP (1:3000, Z0334, DAKO) or anti-Iba1 (1:1000, CP 290B, Biocare Medical) antisera diluted in PBS containing 1% normal serum and 0.5% Triton X-100. The primary antibodies were visualized by the avidin-biotin-complex method (Vectastain Elite ABC Kit, Vector Laboratories). The spinal cord sections were rinsed in PBS (5 × 10 min), incubated in the secondary biotinylated antibody for 1 h and immediately placed in AB-complex for 30 min. After rinsing with PBS (5 × 10 min), the peroxidase was visualized with 0.03% 3,3’-diaminobenzidine tetrahydrochloride (DAB, Sigma) activated with 0.01% H_2_O_2_. The reaction was stopped with distilled water and excess reaction product was washed out with PB. The sections were dried overnight and then dehydrated through ascending concentrations of alcohol, followed by three changes of xylene. Finally, the slides were coverslipped using DePex (Serva, Germany).

### Quantitative microscopy

Five immunostained spinal cord sections per slide were randomly selected on an AxioImager D1 microscope (Carl Zeiss) and photographed (AxioCam MRm, Carl Zeiss). Using a software tool (AxioVision, Carl Zeiss) a mask representing the boundaries of the dorsal horn (lamina I to V) was drawn for further image analysis. To differentiate background staining from true positive immunostaining statistical clues were used as described by Hoheisel et al. [43]. For each section the grey values of three randomly chosen unstained areas were measured and the mean was defined as background. The threshold level for “true” immunostaining was then set as the background value plus three standard deviations. Afterwards, the software was able to compute the ratio (in percentage) of immunostained area to total area (=area of the mask), also expressed as staining density.

We did not detect any significant differences in the staining density of the left side of the spinal cord both when comparing the three strains and the control versus CCI conditions. This allowed us to consider the left dorsal horn (contralateral to CCI) as control and to set the respective staining density as 100%. The % change for the right dorsal horn (ipsilateral to CCI) was calculated for each spinal level of each rat. Finally, the mean per level per strain was calculated.

### Sample collection and RNA isolation

At day 4 or 21 post surgery the 12 animals per strain per time point (6 sham and 6 CCI each) selected for RNA isolation were anesthetized with isoflurane and decapitated. Levels L4/L5 of the spinal cord were removed and ipsi- and contra-lateral sides were separated. Total RNA was extracted using the Invitrap Spin Tissue RNA Microkit (Invitek, Berlin, Germany). The total RNA concentration was determined by measuring the absorbance at 260 nm, using a Nanodrop ND-2000 spectrophotometer (Thermo Fisher Scientific, Wilmington, USA).

### Reverse transcription and real-time qPCR

Total RNA (500 ng) was reverse transcribed into cDNA using the ImProm-II Reverse Transcription System (Promega Corporation, Madison, USA). Real-Time PCR reactions were performed from 10 ng of cDNA with a CFX-96 thermocycler (Bio-Rad Laboratories, Nazareth, Belgium). SYBR green Supermix PerfeCTa (95053-02 K, Quanta Biosciences, Gaithersburg, MD, USA) was used for the detection of Iba1, GFAP, EAAT2, EAAT3, and β-actin (as internal reference). Primer sequences are listed in Table 1. The steps consisted of one cycle of 3 min at 95°C and 40 cycles of amplification (10 sec at 95°C, 30 sec at 61°C). All samples were run in triplicate. Relative expression was estimated using the standard ΔΔCT-method published by Pfaffl [44]. In this mathematical model threshold cycle values (CT) were used to compute the amount of target gene mRNA in relation to the reference gene mRNA (β-actin). ΔCT represents the difference between the number of cycles that were necessary to detect the PCR products of the target and the reference genes. The ΔΔCT indicates the difference between the ΔCT of the neuropathic group and the ΔCT of the sham-operated (control) animals and reflects the extent of fold difference of under- or overexpression of the gene of interest in the CCI animals relative to the sham-operated control group (relative expression level of control?=?1). The data were expressed as 2^-ΔΔCT^. For each strain the mean was computed.

**Table 1:** Sequences of primers used in this study

**Name**	**Accession**	**Sequence**
β-actin	NM_031144	5′ GCT GAG AGG GAA ATC GTG CGT GAC 3′
		5′ GGA GGA AGA GGA TGC GGC AGT GG 3′
Iba1	NM_017196	5′ TCC CAT CCA ACC TCT CTT CC 3′
		5′ GCA GCC TCA TCG TCA TCT C 3′
GFAP	NM_017009	5′ TGA GTC GCT GGA GGA GGA G 3′
		5′ GCT GTG AGG TCT GGC TTG G 3′
EAAT3	NM_013032.3	5′ TCA TAG TCG GGA AGA AC 3′
		5′ AGC GGA ATG TAA CTG GAA GG 3′
EAAT2	NM_017215	5′ ATG CTC CTC ATT CTC ACA G 3′
	NM_001035233	5′ CTA CAT TGA CCG AAG TTC TC 3′

### Statistical analysis

Statistical analyses for mechanical thresholds and corticosterone levels were carried out using an analysis of variance (ANOVA) followed by a Scheffé *post hoc* test to check for differences between strains. Comparisons of mechanical thresholds between sham and CCI groups of each strain were performed with a student’s t-test. PCR and immunohistochemical data were analyzed using ANOVA followed by a Tukey-HSD *post hoc* test. P < 0.05 was defined as the level of statistical significance. Statistical tests were performed with IBM SPSS Statistics version 19 (IBM corporation, Somers, NY, USA).

## Results

### Effect of CCI surgery on mechanical pain thresholds in LEW, FIS and WIS rats

On three consecutive days before performing CCI or sham surgery LEW, FIS, and WIS rats were tested for baseline mechanical paw withdrawal thresholds. The average threshold of the right hind paw was 111.83 ± 4.54 mN for LEW, 91.85 ± 5.84 mN for FIS, and 90.72 ± 5.76 mN for WIS rats. Statistical analysis revealed significant differences in mechanical sensitivity between LEW and FIS (p?=?0.032) and between LEW and WIS rats (p?=?0.020). Whereas FIS and WIS did not differ in their withdrawal latency before surgery, LEW was less sensitive. In order to assess the effects of sciatic nerve injury on pain behavior in the individual strains, we calculated the pain thresholds at the different time points in relation to baseline, which was set to 100%.

In the sham operated (control) groups mechanical pain thresholds slightly decreased in all three strains. The paw withdrawal latency in WIS rats descended to 84.01 ± 6.60% of baseline 4 days after sham surgery and subsequently recovered at d14 (d7: 85.57 ± 5.44; d10: 90.27 ± 5.93; d14: 99.81 ± 9.95; d21: 94.49 ± 8.81). In LEW rats the mechanical pain threshold decreased to 78.15 ± 6.71% of baseline at d4 and slightly increased until d21 (d7: 79.09 ± 5.57; d10: 88.30 ± 3.76; d14: 72.36 ± 4.18; d21: 85.51 ± 7.12). FIS rats proved to be more sensitive to the sham operation compared to LEW and WIS. Their paw withdrawal latency dropped to 73.94 ± 5.62% of baseline on d4 and remained low until the end of the experiment (d7: 63.70 ± 4.22; d10: 58.80 ± 5.64; d14: 70.22 ± 5.88; d21: 53.43 ± 4.46). Statistical analysis (ANOVA followed by Scheffé *post hoc* test) revealed significant differences between the FIS and WIS strains on days 7 to 21 (d7: p?=?0.019; d10: p?=?0.0008; d14: p?=?0.025; d21: p?=?0.0008) and between the FIS and LEW rats on days 10 (p?=?0.002) and 21 (p?=?0.009).

**Figure 1:**
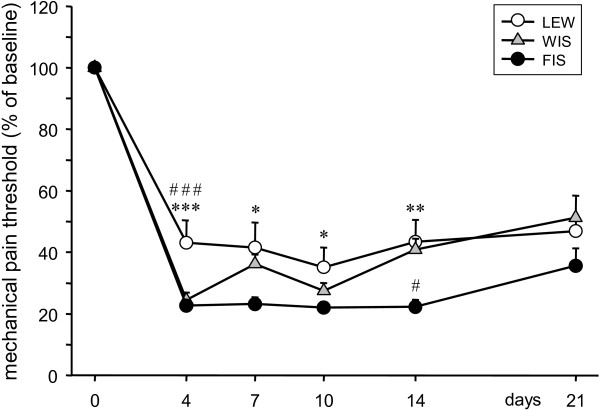
**Effect of CCI on mechanical pain thresholds in LEW, FIS and WIS rats.** The paw withdrawal response to mechanical stimulation of the right hind paw was measured in Lewis (LEW), Fischer (FIS) and Wistar (WIS) rats before and after CCI surgery. For up to 14 days neuropathic pain behavior was less prominent in LEW than in FIS rats. In comparison with the control strain (WIS), LEW was less sensitive in the early phase after nerve injury (d4), whereas FIS showed enhanced sensitivity later on at day 14. Data are shown as mean ± s.e.m. Asterisks represent a significant difference between the LEW and the FIS strain for the individual time point (*p < 0.05, **p < 0.01, ***p < 0.001). ^#^indicates a significant difference between LEW or FIS and WIS (^#^*p* < 0.05, ^###^*p* < 0.001).

After CCI surgery (Figure 1) the mechanical thresholds in LEW rats dropped to 43.12 ± 7.29% of baseline at d4 and remained at that level until the end of the experiment at d21 (d7: 41.54 ± 8.17; d10: 35.12 ± 6.50; d14: 43.43 ± 7.13; d21: 46.87 ± 4.86).

FIS rats displayed a higher mechanical sensitivity at d4 after operation. The paw withdrawal latency descended to 22.63 ± 2.02% of baseline and stayed at that level until d14 (d7: 23.18 ± 2.27, d10: 21.98 ± 1.91; d14: 22.25 ± 2.4). On d21 a slight decrease of the hypersensitivity was observed (35.61 ± 5.71).

In WIS rats the mechanical pain threshold also dropped on d4 following CCI to 24.50 ± 2.48% of baseline. However, there was a slight recovery in the time course of the experiment (d7: 36.18 ± 3.14, d10: 27.46 ± 2.62; d14: 40.86 ± 3.63; d21: 51.25 ± 7.15).

Multiple comparisons revealed that LEW rats remained less sensitive than FIS up to 14 days after the CCI surgery (d4: p?=?0.0003; d7: p?=?0.019; d10: p?=?0.047; d14: p?=?0.0067) and also were less sensitive than WIS on d4 (p?=?0.00096). FIS rats showed no statistical significant difference of threshold values compared to WIS except on d14, where FIS still was more sensitive than the control rats (p?=?0.016).

As expected, sham groups and CCI groups differed significantly in their mechanical pain thresholds at all time points after the surgery, independently of the strain.

### Effects of CCI on plasma corticosterone levels

**Figure 2:**
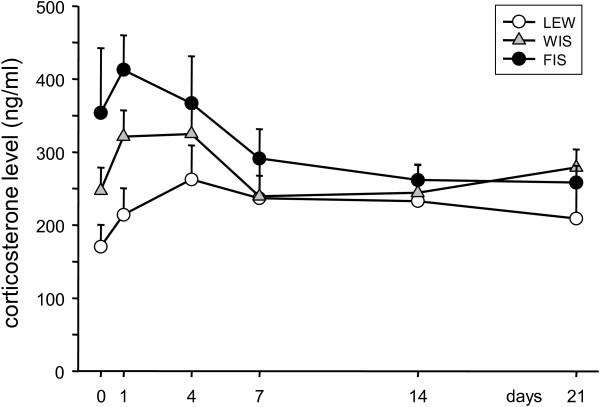
**Time course of serum corticosterone levels in response to CCI of the right sciatic nerve.** Basal corticosterone levels (day 0) were highest in FIS and lowest in LEW rats, however without reaching significance levels due to a high variance in the FIS strain. Induction of neuropathy led to an increase of the corticosterone concentration in all three strains in the first days. The levels subsequently decreased and ended up below baseline values in FIS rats or above in LEW. Data are shown as mean ± s.e.m.

Blood corticosterone levels of the three strains were measured before the surgery (day 0) and at days 1, 4, 7, 14, and 21 following CCI (Figure 2). Before the operation, the corticosterone concentration was lowest in LEW rats (170.5?±?30.0 ng/ml) and highest in FIS (353.7?±?88.7 ng/ml). In the WIS strain the plasma corticosterone level was 247.3?±?31.6 ng/ml and hence was situated between the levels seen in FIS and LEW. However, statistical comparison between the groups did not show significant differences due to a high variation in the FIS strain.

During the development of neuropathy FIS rats reacted first with an increase of the corticosterone level on d1 (412.6?±?47.2 ng/ml). In the course of the following days the concentration did however even decrease below baseline level (d4: 366.8?±?64.4, d7: 291.3?±?40.0, d14: 261.8?±?20.5, and d21: 258.2?±?23.3 ng/ml). In LEW rats the plasma corticosterone increased slowly, peaking at d4 and decreased slightly until the end of the experiment on d21, without reaching the baseline level (d1: 214.2?±?36.5, d4: 262.5?±?46.6, d7: 236.7?±?59.7, d14: 232.6?±?50.6, and d21: 209.0?±?65.6 ng/ml). WIS rats exhibited a slight increase of the corticosterone level following CCI on d1 (321.3?±?36.0 ng/ml) and d4 (324.8?±?34.6 ng/ml) and came back to baseline on d7 (239.3?±?28.3; d14: 244.1?±?20.9, d21: 279.0?±?24.6 ng/ml). Within the strains we did however not observe significant differences in the temporal pattern of corticosterone concentrations compared to day 0 which might be related to high variability levels.

### CCI induced activation of spinal glia cells in LEW, FIS, and WIS rats

In order to characterize the activation of spinal glia cells following the CCI surgery we examined the expression of mRNA and protein of the glia-specific markers Iba1 (microglia) and GFAP (astrocytes) by using qPCR and immunohistochemical techniques.

#### Activation of microglia

**Figure 3:**
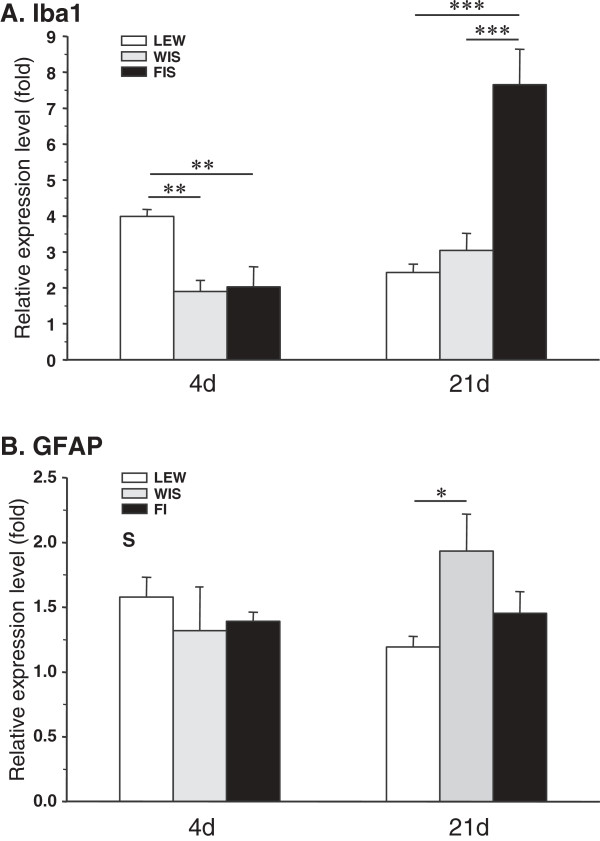
**CCI-mediated mRNA expression of the glial markers Iba1 and GFAP in the three rat strains.** The relative expression level of **(A)** the microglial activation marker Iba1 and **(B)** the activation marker of astrocytes GFAP was measured at the spinal cord level L4/5 of Lewis (LEW), Fischer (FIS) and Wistar (WIS) rats on days 4 and 21 after chronic constriction injury of the right sciatic nerve. Strains only differed in their temporal activation profile of microglia: Lewis rats reacted shortly after CCI with a significant increase of Iba1, whereas Fischer rats displayed a high upregulation of this marker at a later time point. The relative expression level indicates the fold difference of the CCI group to the sham-operated control group (control?=?1). Data are expressed as mean ± s.e.m. *p < 0.05, **p < 0.01, ***p < 0.001.

At days 4 and 21 post surgery total mRNA was isolated from the spinal cord and, after reverse transcription into cDNA, qPCR was performed using a specific primer for Iba1 (Table 1) to detect differences between strains. Four days after CCI the Iba1 mRNA was upregulated in all three strains (Figure 3A). Whereas the relative expression level was twofold in FIS and WIS rats (FIS: 2.03?±?0.56; WIS: 1.90?±?0.31), we measured a fourfold increase in LEW (3.98?±?0.20). This situation reversed in the course of the neuropathic state: 21 days after CCI surgery the relative expression level of Iba1 decreased to 2.43?±?0.23 in LEW rats, but increased in WIS (3.04?±?0.48) and even more in FIS (7.65?±?0.48) (Figure 3A). At d4 comparison between groups (ANOVA followed by Tukey-HSD *post hoc* test) revealed a significant increase of Iba1 mRNA expression in the LEW strain compared to FIS and WIS (LEW vs. FIS: p?=?0.007; LEW vs. WIS: p?=?0.003) and at d21 a significantly higher expression level was observed in FIS compared to LEW and WIS rats (FIS vs. LEW: p?=?0.0002; FIS vs. WIS: p?=?0.0008).

**Figure 4:**
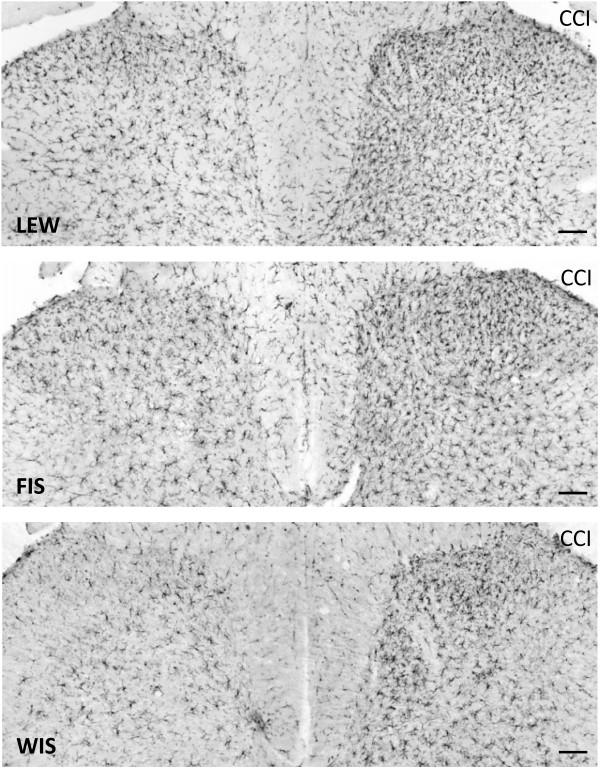
**Iba1-immunostaining in LEW, FIS and WIS rats subjected to nerve injury.** The photomicrographs show the effect of a chronic constriction injury (CCI) of the right sciatic nerve on microglial cell activation in the dorsal horn of the spinal cord segment L4 on day 21 after surgery in Lewis (LEW; Top), Fischer (FIS; Middle) and Wistar (WIS; Bottom) rats. The number and staining density of Iba1-immunopositive microglial cells was clearly enhanced in the right dorsal horn (side of CCI) compared to the left unaffected side in all three strains. Differences between strains were not obvious. Scale bars: 100 μm.

**Figure 5:**
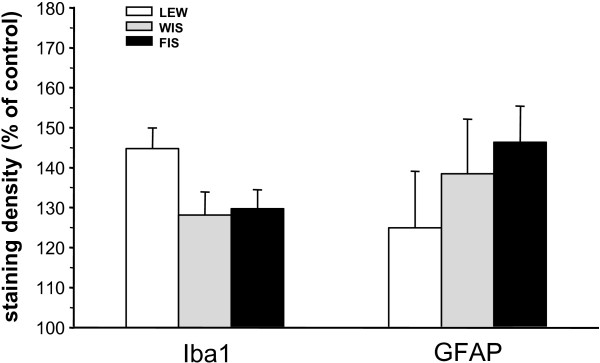
**Staining density of the glial markers Iba1 and GFAP at day 21 post-CCI.** The immunohistochemical staining density of microglial (Iba1) and astrocytic (GFAP) activation markers in the dorsal horn of the spinal cord segments L4/L5 was quantified 21 days after induction of chronic constriction injury (CCI) in Lewis (LEW), Fischer (FIS) and Wistar (WIS) rats. Iba1- and GFAP-immunostaining was enhanced on the side of CCI in relation to the left unaffected side (control = 100%) in all three strains. The staining density of the microglial marker Iba1 seemed to be higher in LEW rats compared to FIS and WIS but this difference did not reach significance. No differences between strains could be found for the expression of GFAP protein. Data are shown as mean ± s.e.m.

At day 21 post-operation, a CCI-related activation of microglia was detected at the ipsilateral spinal cord dorsal horn levels L4/L5 of all three strains. The glial cells were intensely immunostained for Iba1 and their cellular processes appeared shorter and thicker than those in the contralateral dorsal horn. We observed an increase in the staining density of the Iba1 protein in the right dorsal horn (side of surgery) compared to the left (unaffected) side in all three strains (Figure 4). Although the staining density was obviously higher in LEW rats (144.7 ± 5.2%) (Figure 5), the statistical test failed to show significant differences for the FIS (129.7 ± 5.2%) and WIS (128.1 ± 6.4%) strain.

Taken together, LEW and FIS displayed a differential time course of microglia reaction. In LEW rats the activation of microglia was most pronounced within the first days after nerve constriction and subsided in the course of persistent neuropathy. In contrast, FIS displayed a significantly delayed CCI-related considerable raise in Iba1 mRNA expression.

#### Activation of astrocytes

Four and 21 days after CCI surgery we harvested the spinal cord level L4/L5 and carried out qPCR by using a specific primer for GFAP (Table 1) to detect differences between strains. At d4 the relative expression level of GFAP mRNA was slightly enhanced in all three strains (LEW: 1.58?±?0.15; FIS: 1.39?±?0.07; WIS: 1.32?±?0.34) (Figure 3B). Although the mRNA expression tended to be higher in LEW rats, the statistical evaluation did not yield significant differences in comparison with FIS and WIS animals. At 21 days post-operation the GFAP mRNA level decreased in LEW (1.19?±?0.08), remained constant in FIS (1.45?±?0.17), but significantly increased in WIS (1.93?±?0.29; WIS vs. LEW: p?=?0.038) (Figure 3B). Again, the LEW strain seemed to display an earlier activation of this type of glia cells. However, in contrast to microglia, astrocytes were not highly activated in FIS rats at later stages of the nerve injury.

**Figure 6:**
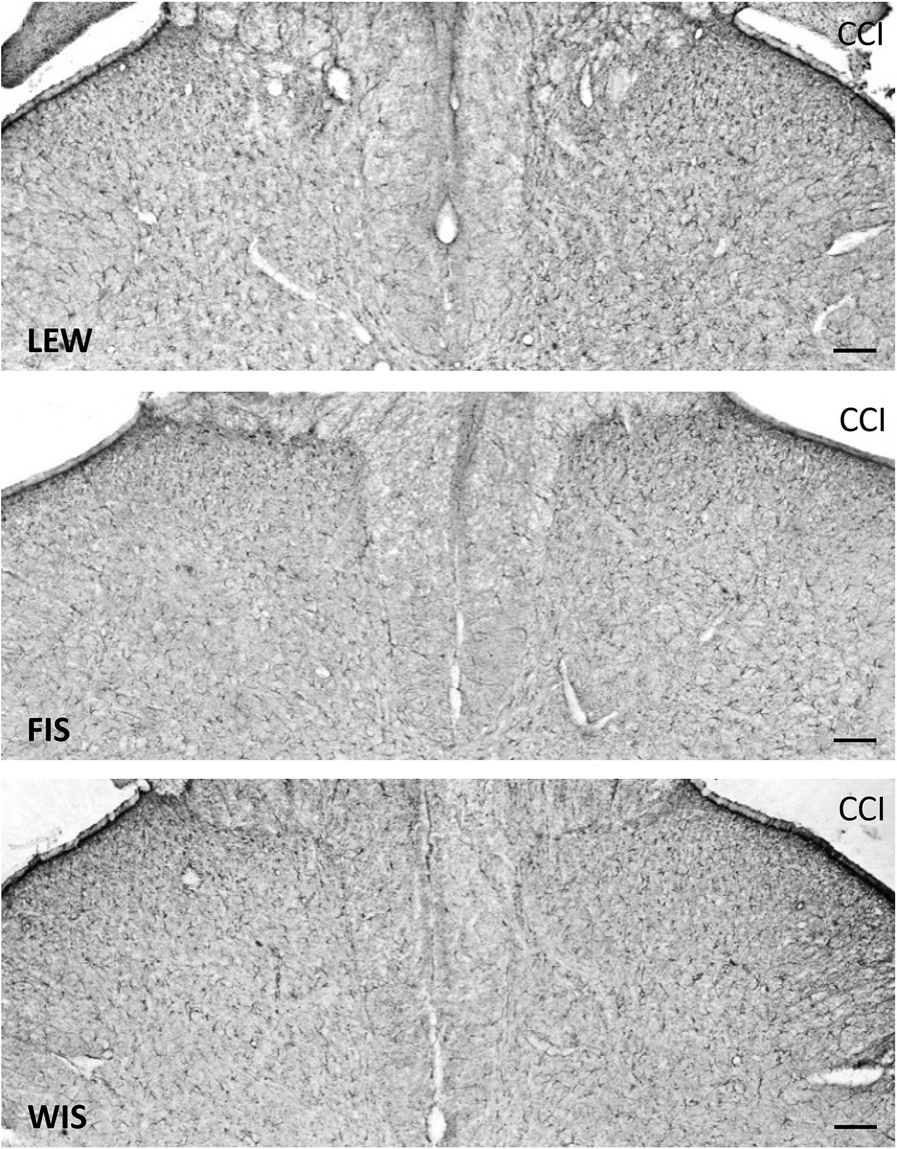
**GFAP-immunostaining in LEW, FIS and WIS rats subjected to nerve injury.** The photomicrographs show the effect of a chronic constriction injury (CCI) of the right sciatic nerve on the activation of astrocytes in the dorsal horn of the spinal cord segment L4 at day 21 after surgery in Lewis (LEW; Top), Fischer (FIS; Middle) and Wistar (WIS; Bottom) rats. The number and staining density of GFAP-immunopositive astrocytic cells was enhanced in the right dorsal horn (side of CCI) compared to the left unaffected side in all three strains. Significant differences between strains were not observed. Scale bars: 100 μm.

The immunohistochemical staining of the astrocytic marker GFAP in the spinal cord sections revealed a change in the morphology of these glial cells at day 21 after CCI surgery. The GFAP immunostained astroglial cells appeared hypertrophic in the right dorsal horn of the spinal cord. Additionally, we observed an increase in the staining density of the GFAP protein in the right dorsal horn (side of nerve injury) compared to the left not affected side in all three strains (Figure 6). Statistically significant differences between the strains could not be obtained due to high variations within the groups (LEW: 125.0?±?14.1; FIS: 146.3?±?10.0; WIS: 138.4?±?15.0%) (Figure 5).

### Modification of neuronal and glial glutamate transporter mRNA expression following CCI

Glutamate transporters control the availability of glutamate in the synaptic cleft and thus regulate the activation of glutamate receptors. EAAT3 (also known as EAAC1) is associated with neurons, whereas EAAT2 is expressed in glia cells. Here we used qPCR to assess nerve injury-related differences in the regulation of the neuronal and glial glutamate transporter expression in the three rat strains.

#### Expression of EAAT3

**Figure 7:**
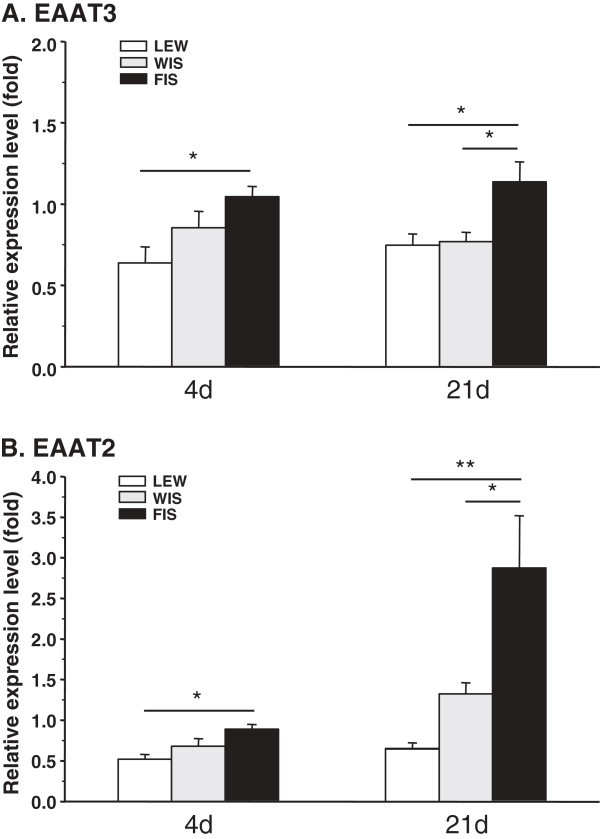
**CCI-mediated mRNA expression of spinal EAATs in LEW, FIS and WIS rats.** The relative expression level of **(A)** the neuronal (EAAT3) and **(B)** the glial glutamate transporter (EAAT2) was determined in the spinal cord level L4/5 of Lewis (LEW), Fischer (FIS) and Wistar (WIS) rats at days 4 and 21 after chronic constriction injury of the right sciatic nerve. **(A)** EAAT3 mRNA was downregulated in the LEW strain already after four days and still after 21 days, in WIS only after 21 days, but was unchanged in FIS. **(B)** EAAT2 mRNA expression was also decreased in LEW rats at d4 and d21. In WIS it was first downregulated but increased after 21 days and in the FIS strain the glial glutamate transporter was nearly unchanged early after surgery but highly upregulated at the later time point. The relative expression level indicates the fold difference of the CCI group to the sham-operated control group (control?=?1). Data are expressed as mean ± s.e.m. *p < 0.05, **p < 0.01.

Four days after CCI surgery the neuronal glutamate transporter was clearly downregulated in LEW rats (0.64?±?0.10), to a lesser extent in WIS (0.85?±?0.10), but unaffected in FIS (1.05?±?0.06) (Figure 7A). At that time point LEW significantly differed from the FIS strain (p?=?0.031).

In the course of neuropathy the relative expression level of the neuronal transporter only changed slightly. At day 21 LEW and WIS rats still exhibited less EAAT3 mRNA (LEW: 0.75?±?0.07; WIS: 0.77?±?0.06) whereas mRNA levels tended to increase in FIS (1.14?±?0.12) (Figure 7A). Significant differences were obtained between FIS and LEW rats (p?=?0.019) and between FIS and WIS (p?=?0.034).

#### Expression of EAAT2

Shortly after the CCI operation (d4) the glial glutamate transporter also was clearly downregulated in LEW (0.52?±?0.06), to a lesser extent in WIS (0.68?±?0.09) and only slightly in FIS (0.89?±?0.06) animals (Figure 7B). The statistical analysis revealed a significant difference between the LEW and the FIS strain (p?=?0.015). At d21 post surgery the EAAT2 mRNA expression level was still down in the LEW group (0.66?±?0.06), there was however a tendency towards enhancement. In WIS rats the early decrease turned to an increase (1.32?±?0.14) and in FIS a nearly threefold upregulation (2.87?±?0.64) could be observed at that late time point (Figure 7B). The differences between the FIS and the two other groups are statistically significant (FIS vs. LEW: p?=?0.004; FIS vs. WIS: p?=?0.046). Taken together, the LEW strain reacted with an early downregulation of the neuronal as well as the glial glutamate transporter. For both markers, a slight tendency towards recovery was after 21 days. In contrast, FIS rats only displayed a clear modulation of the glial glutamate transporter mRNA expression at the later time point. This temporal reaction pattern is reminiscent with the time course of glia activation observed in the two rat strains.

## Discussion

In the present study, we aimed to determine whether the differential HPA axis reactivity expressed by Lewis and Fischer rats has an impact on pain sensitivity, glial cell activation and on the regulation of the expression of glutamate transporters in a context of neuropathy. Following CCI surgery, we observed that LEW rats were less sensitive than FIS up to 14 days post-operation while the two strains did not display any significant difference in their plasma corticosterone levels. Regarding glial activation and the expression of glutamate transporters, we found different temporal transcription patterns between LEW and FIS rats.

### Development and maintenance of mechanical allodynia

Nociceptive sensitivity tests from the fourth day post-surgery demonstrated that CCI effectively induced mechanical allodynia in the three rat strains. Surprisingly, LEW rats exhibited less pain sensitivity from days 4 to 14 after surgery, as compared to the other strains. This result is contradictory to the findings of Fecho and Valtschanoff [16] stating that LEW rats present an exacerbated mechanical allodynia compared to FIS rats after partial ligation of the sciatic nerve. This discrepancy may result from differences in the chosen models of neuropathic pain and the inherent surgical procedures. The paradigm used in the present study may be accompanied by a more significant injury and concomitant inflammatory insult that may in turn (at least initially) lead to a more pronounced pain sensitivity in FIS rats [45,46]. This interpretation seems to be corroborated by the levels of allodynia also expressed by the sham operated FIS rats. Other investigators did not observe any differences between LEW and FIS undergoing neuropathic pain [47,48]. Nevertheless, in accordance with our results, LEW rats have also been described to be less sensitive than FIS intrinsically or shortly following peripheral inflammation [45,46,49,50]. Altered HPA-axis reactivity may hence in fact have an impact on pain sensitivity.

It is well known that FIS rats display significantly higher basal and stress-related corticosterone levels than LEW rats [10,13,51,52]. To our knowledge, no studies exist about the long-lasting pain-related evolution of corticosterone levels in LEW and FIS rats. Although we failed to observe any significant differences, FIS, LEW and WIS seemed to display different patterns of progression for this parameter. FIS displayed a lower level of plasma corticosterone at day 21 whereas LEW exhibited a higher level at this time point. The fact that LEW rats were less pain sensitive at day 4 after CCI induction in spite of showing lower plasma glucocorticoid levels compared to FIS and WIS rats may not seem consistent with the expected protective anti-inflammatory effects of corticosteroids. Under the present conditions circulating corticosterone may thus not be a major etiological factor explaining the observed pain sensitivity discrepancies of LEW rats compared to the other strains. Additional factors to be taken into consideration might be related to strain-specific regulations of glutamatergic transmission and/or of the glial cell activation in the spinal cord.

### Glial cell activation

Microglial and astrocytic activation is involved in the initiation and maintenance of sensitivity in neuropathic or pathological pain [27-29,36,53-56]. It has been demonstrated that peripheral nerve injury leads to an activation of spinal microglia as characterized by morphological modifications of these cells and by an enhanced expression of markers such as OX-42 and Iba-1 [34]. Our results showed a time-dependent difference in the activation pattern of microglia between LEW and FIS rats. In fact, 4 days after CCI, Iba-1 mRNA expression was upregulated in LEW rats whereas in FIS an upregulation was only seen after 21 days. At the same time Iba-1 protein levels however tended to be upregulated in all strains. This effect seemed to be somewhat more pronounced in LEW rats, even though the observed alterations were not significant. At a first glance, the data from day 21 seem to suggest a major discrepancy between the enhanced relative expression of Iba-1 mRNA and the lack of a concomitant elevation of the respective (immunohistochemically determined) protein level in the FIS rats (Figures 3A and 5). Since the elevated mRNA expression could only be observed in this late phase of neuropathy it is possible that the translation into protein had not yet been significantly implemented (at this time point of observation). Alternatively, the described mismatch could have resulted from unidentified regulatory mechanisms or from an activity-dependent increase in the turnover of the protein as discussed by Mistry and colleagues [57] who described similar findings for TRPV1 mRNA and protein expression in rat spinal ganglion neurons.

Since the different strains of rats did not display any differences in mechanical allodynia at this time point, we were not able to establish any relation between Iba-1 expression and pain sensitivity, contrary to the investigations of Romero-Sandoval and colleagues [58-60] and other studies concluding that an inhibition of microglial activation reduces the development of neuropathic pain [27,61-63]. In the present conditions microglial activation was hence again not sufficient to explain the observed differences in pain sensitivity.

On the other hand, the expression of the astrocytic marker GFAP was not altered at day 4 after CCI. This is in agreement with the assumption that microglia is the glial cell type initially responding to nerve injury [37,64,65]. The importance of astrocyte activation for the maintenance of neuropathic pain is supported by studies from Romero-Sandoval and colleagues [58,60]. Four days after L5 nerve transection these investigators did not observe any robust change of GFAP expression at the protein level. Nevertheless, it has been recently shown that neuropathic pain induces an upregulation of GFAP expression in the spinal cord from day 3 or day 4 after spinal nerve ligation or L5 spinal nerve transection [29,35]. Astrocytic activation persists throughout the duration of neuropathic pain while microglial activation may have declined again [66]. Although LEW tended to express less GFAP than FIS rats, we failed to demonstrate any significant upregulation or difference in GFAP expression between the two strains at day 21, either at the transcript or at the protein levels. A CCI-related activation of astrocytes has however been demonstrated by Garrison and colleagues [30].

As already discussed, astrocytes seem to be responsible for the maintenance of hypersensitivity following peripheral nerve injury [27,53,67] whereas microglia rather seem to be involved in the induction of neuropathic pain [34,68]. Additional mechanisms of pain regulation may however have to be considered. Recent studies have e.g. described the involvement of perivascular microglia in inflammation and peripheral nerve injury. These antigen-presenting cells reside in close association with astrocytes and endothelial cells and express ED2 (ED2/CD163 scavenger receptor cysteine-rich group B family member) [69], a molecule associated with anti-inflammatory processes [70,71]. It has been shown that a L5 spinal nerve transection reduces ED2 expression, whereas a cannabinoid receptor 2 agonist enhances spinal ED2 expression and inhibits tactile allodynia [59]. These findings suggest that ED2 expressing perivascular microglia may have a role in the processing of neuropathic pain. This subtype of microglia could thus be differentially involved in the setting of mechanical allodynia/hyperalgesia in LEW and FIS rats, a factor that could contribute to the explanation of the discrepancies in the respective pain thresholds.

Concerning our immunohistochemical assessment of Iba-1 and GFAP protein expression, one point needs further discussion. The fact that we used the side contralateral to the CCI as control seems to imply that we did not consider the potential development of mirror-image neuropathic pain resulting from the activation of glia cells [28]. Bilateral labeling of activated microglia in the spinal cord dorsal horn following a unilateral spared nerve injury has e.g. been described in rats [72]. In the present study, the three tested strains also displayed contralateral albeit less pronounced Iba-1 and GFAP immunoreactivity as compared to the treated side. These lower levels of protein expression are not necessarily accompanied by an increase in pain sensitivity. In all animal groups we did in fact observe stable behavioral responses to contralateral paw stimulation. This lack of alterations may be related to a time dependent dissociation between pain behavior and the measured biochemical parameters (see [73]). It is also possible that a significantly more pronounced ipsilateral expression of spinal mediators developed in the initial phase preceding day 4 after CCI and then gradually decayed, tending to converge with the respective values of the untreated side. Taken together, the constancy of responses to stimulation of the non-treated side allowed us to consider this side as a reference for the elucidation of CCI-mediated effects.

### Glutamate transporters

It is well known that the major part of glutamate released in the synaptic cleft is removed by surrounding astrocytes. The EAATs are located in the plasma membrane of these cells and maintain the extracellular glutamate concentration in the physiological range. EAATs are known to play a critical role in both induction and maintenance of neuropathic pain induced by peripheral nerve injury [24,74]. In this framework, glucocorticoids (GCs) as well as their receptors have also been shown to be implicated. After peripheral nerve injury, Wang and colleagues [26] have demonstrated a negative regulation of EAAT3 through activated spinal GC receptors and a concomitant positive regulation of the NMDA receptors, highlighting the synergetic role of these two systems in the enhancement of pain. In our study, EAAT2 and 3 were only downregulated 4 days post-surgery in the spinal cord of LEW rats; no modification was observed in FIS and in WIS rats. After 21 days, we could again notice a decreased expression of glutamate transporters in LEW, but also an increased expression of EAAT2 in FIS. This is inconsistent with previous studies demonstrating that CCI or inflammation causes a rapid transient upregulation of EAATs in the spinal cord, followed by a downregulation [24]. It should however be kept in mind that we only studied transcript expression levels of EAATs and that post-transcriptional regulation could have led to different protein levels of the glutamate transporters.

Taken together, the results we obtained with spinal markers are conflicting: 21 days after CCI, we observed an upregulation of Iba-1 mRNA in FIS rats, which should be accompanied by pain hypersensitivity. Concomitantly, we demonstrated an upregulation of EAAT2, which normally triggers a reduction of the sensitivity [24]. These opposing processes might have counterbalanced each other, leading to a relative reduction of pain sensitivity in FIS rats. In LEW rats, the documented modifications of Iba-1 and EAATs should have led to mechanical hyperalgesia [21,58,60,75]. We noticed the contrary since LEW turned out to be the least sensitive to pain. This points again to the fact that under the conditions of the present study glial markers may not constitute reliable indicators of pain sensitivity.

One final issue should be discussed with respect to selecting day 21 as the only time point for the measurement of biochemical parameters in the later phase of neuropathy. The lack of differences in CCI-related mechanical allodynia between the strains at that time point suggests that additional measurements at shorter intervals might have been required to follow the temporal relations between pain behavior and the expression of biochemical markers in a more consistent way.

## Conclusion

The complex network of pathways required for the development and the maintenance of neuropathic pain [18] clearly exceeds the simple corticosterone level, glial markers or glutamate transporters expression, which were insufficient to explain the differences in pain processing between LEW, FIS and WIS rat strains. In this context, it should be kept in mind that although the differential HPA axis reactivity of LEW and FIS rats provides a widely accepted comparative model for investigating interactions between nervous, endocrine and immune systems [10], these inbred strains also display significant differences in gene expression [76] as well as in cardiovascular and behavioral reactivity to stress [77,78] that may impede the interpretation of our findings. Pharmacological studies based on manipulation of the HPA axis (glucocorticoid receptor agonists and antagonists, different doses and time courses) in a common rat strain will be required to gain further insight into its potential implication in neuropathic pain. Finally, it will be necessary to consider that supraspinal e.g. limbic and descending pain control networks may play a critical role [18,79,80].

## Abbreviations

HPA axis: Hypothalamo-pituitary-adrenal axis; CCI: Chronic constriction injury; LEW: Lewis rats; FIS: Fischer 344 rats; WIS: Wistar rats; GFAP: Glial fibrillary acidic protein; Iba1: Ionized calcium-binding adapter molecule 1; EAAT3: Excitatory amino acid transporter 3; EAAT2: Excitatory amino acid transporter 2; CRH: Corticotropin-releasing hormone; ACTH: Adrenocorticotropic hormone; mRNA: Messenger RNA; qPCR: Quantitative PCR; EDTA: Ethylenediaminetetraacetic acid; ELISA: Enzyme-linked immunosorbent assay; PBS: Phosphate-buffered saline; PB: Phosphate buffer; DAB: 3,3’-diaminobenzidine tetrahydrochloride; CT: Threshold cycle values; RIA: Radioimmunoassay; ED2: ED2/CD163 scavenger receptor cysteine-rich group B family member; GC: Glucocorticoid; NMDA receptor: N-methyl-D-aspartate receptor

## Competing interests

The authors declare that they have no competing interests.

## Author’s contributions

Conception and study design: FA, UH. Performing of experiments and acquisition of data: G-MLC, CF. Analysis and interpretation of data: G-MLC, CF, FA, UH. Writing the manuscript: G-MLC, CF, FA, UH. All authors read and approved the final manuscript.
